# Necrotizing Fasciitis Presenting as an Itchy Thigh

**DOI:** 10.1155/2016/6376301

**Published:** 2016-08-04

**Authors:** Thor Shiva Stead, V. Shushrutha Hedna

**Affiliations:** ^1^Trinity Preparatory High School, 5700 Trinity Prep Ln, Winter Park, FL 32792, USA; ^2^University of New Mexico, Albuquerque, NM 87131, USA

## Abstract

We present a case of necrotizing fasciitis diagnosed in the emergency department. The clinical presentation and diagnostic findings of necrotizing fasciitis are discussed.

## 1. Introduction

Necrotizing fasciitis is a severe soft tissue infection which, left untreated, is usually lethal. Although relatively rare, it is more common in diabetics, who also have poorer prognosis [1]. Emergency physicians should maintain a high index of suspicion particularly in diabetics and the immunocompromised ones. Dissecting gas along fascial planes in the absence of penetrating trauma is pathognomonic [2].

## 2. Case Presentation

A 53-year-old obese male presented due to a wound in his right thigh. He stated that the wound started as an itch and that it progressed to its current state in just 5 days. The wound started to smell bad. The patient had a history of diabetes, which he reports to be diet controlled. He is a truck driver by trade and says he does his best to do a good job with his diet. His past medical history was notable for testicular cancer status after radiation 9 years earlier. He states that his left groin skin has always been a little sensitive after the radiation and in that he often will itch it. He says this wound started out as an itch as well. Intense itching led to an open wound, at which point he applied povidone iodine and put a dressing on it. He was remarkably stoic, considering the extent of the wound.

Review of systems is negative except as noted above. The patient specifically denied fever, chills, chest pain, shortness of breath, abdominal pain, nausea, vomiting, diarrhea, urinary symptoms, or headache. His prescribed medications included atorvastatin for hyperlipidemia, Diltiazem and Lisinopril for hypertension, Metformin for diabetes, and Gabapentin for diabetic neuropathy. The patient had no allergies and his tetanus status was up to date.

**Figure 1:**
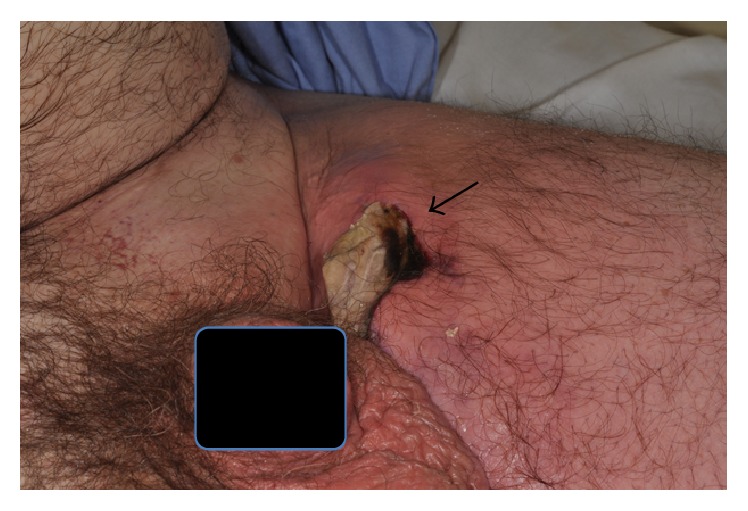
Photograph demonstrating 4 × 10 am elliptical area of necrotic tissue (arrow).

Patient's vital signs were as follows: blood pressure 145/85 mmHg; respiratory rate 20/min; pulse 91/min, SpO_2_ 99%, temperature 36.7°C; and pain 8/10. On physical exam, he was alert and oriented to person, time, and place and in no acute distress. Pertinent positive findings included a 4 cm × 10 cm elliptical wound on the left thigh with necrotic flesh within, which was exquisitely foul smelling (Figure 1). The left leg appeared somewhat mottled and cellulitic compared to right leg. Both legs had intact dorsalis pedis and popliteal pulses.

Laboratory analysis revealed an elevated white blood cell (WBC) count of 17.1 k/cm with the remainder of the CBC within normal limits. The metabolic panel revealed blood sugar of 398 and corresponding hyponatremia of 128 mmol/L. The chloride and bicarbonate were also low at 89 mmol/L and 21 mmol/L, respectively, yielding an elevated anion gap of 18. The patient also has had hypoalbuminemia with a value of 2.5 g/dL. Lactate was elevated at 2.8 mmol/L. Coagulation studies were within normal limits. Blood cultures were sent. The urinalysis demonstrated clear glycosuria with >500 mg/dL of glucose, as well as pyuria with 17 WBC per high power field. There was no ketonuria, leukoesterase, or elevated nitrates.

**Figure 2:**
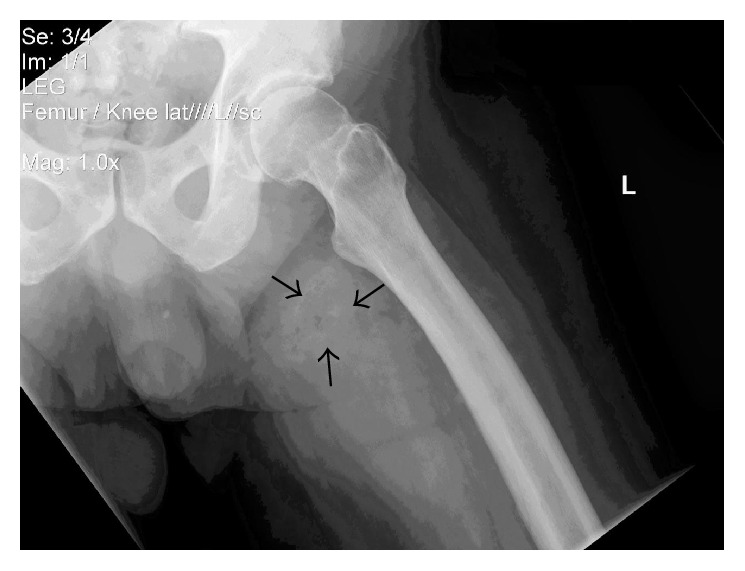
Radiograph of left leg demonstrating gas within the necrotic wound (arrows).

AP and lateral radiographs of the left femur revealed mottled lucencies consistent with gas in the soft tissues of the medial thigh (Figure 2).

A duplex Doppler exam with real time grey-scale imaging, spectral Doppler with wave form analysis, color Doppler, and physiologic maneuvers including compression were done to evaluate the venous system of the left leg from the groin down through the popliteal fossa. Subcutaneous edema was noted in left thigh, but there was no sonographic evidence of deep venous thrombosis.

The patient's presentation was most concerning for gas gangrene versus necrotizing fasciitis. Patient looked remarkably well, despite the differential diagnosis. He was resuscitated with 4 L of NSS, 10 U of intravenous regular insulin, 1 g of intravenous Vancomycin, and 3.375 g of intravenous Piperacillin-Tazobactam. He was given 2 oxycodone tablets for analgesia. Patient's mentation remained intact throughout. The patient was then transferred to the critical care unit where he underwent surgical debridement followed by hospitalization for intravenous antibiotics. He also received diabetes education, as he was not taking his Metformin, but rather under the impression that he was doing a good job controlling his diabetes with diet. His decompensated diabetes was likely a contributing cause to his necrotizing fasciitis. Following hospitalization, the patient was discharged with complete resolution of the infection and left with blood sugar well controlled.

## 3. Discussion

Necrotizing fasciitis is a bacterial, or in rare cases, fungal infection of the subcutaneous layer of the skin. Commonly tossed around as “flesh-eating bacteria,” this deadly disease is typically caused by a swarm of bacteria from the genus* Streptococcus* [3], which are also responsible for common illnesses such as strep throat and rheumatic fever. Upon entering the body, bacteria spread rapidly and infect a layer of skin known as the fascia, which is a flat membrane consisting of connective bands of tissue that surround muscles, nerves, fat, and blood vessels. The thicker this fascia, the higher the rate of necrosis; that is, they are directly proportional. As the infection grows, the bacteria can infect surrounding layers of the skin, even making its way up to the epidermis. The danger lies in the fact that these bacteria produce toxins, which can cause the death of cells, leading to full-blown necrosis. Much like tetanus, the disease is rarely spread from person to person and is most commonly obtained through a break in the skin or puncture wound from foreign object. First origins of the disease date back to 1871, when confederate army surgeon Joseph Jones described “flesh-eating bacteria” in a soldier's leg [4]. Although public outbreaks of necrotizing fasciitis have been on the rise since the 19th century, it is still most common in military personnel and veterans due to the high incidence of physical trauma and puncture wounds. Initial symptoms of this illness include inflammation, redness of the afflicted area, pain greater than expected for visible lesions, fever, and chills. Some individuals may initially complain of pain or soreness, similar to that of a “pulled muscle.” Over the course of hours to days, patients may present with a dark patch of skin that often smells malodorous. This lifeless pool of skin may not even be apparent to the patient, providing enough nerve damage is sustained. If left untreated, continued spread of the infection and widespread bodily involvement invariably occur, frequently leading to sepsis and often death [5]. Therefore, it is imperative that necrotizing fasciitis should be diagnosed quickly and accurately and not dismissed as dermatitis or an allergic reaction. Treatment of this malady most commonly involves surgical removal of the dead tissue and intravenous antibiotics. Prognosis for patients who are diagnosed early is usually favorable; however, the disease can snowball and, if not diagnosed initially, can progress to necessary amputation within a matter of days [6].

The incidence of necrotizing fasciitis is 2.1 per million in the United States [7]. Risk factors for developing the condition include diabetes, drug use, obesity, immunosuppression, recent surgery, and traumatic wounds [8].
